# High sensitive detection of circulating tumor cell by multimarker lipid magnetic nanoparticles and clinical verifications

**DOI:** 10.1186/s12951-019-0548-1

**Published:** 2019-11-25

**Authors:** Jingde Chen, Lin Chen, Shibin Du, Jing Wu, Ming Quan, Hua Yin, Yin Wu, Xuanting Ye, Xiaofei Liang, Hong Jiang

**Affiliations:** 1 Seventh People’s Hospital of Shanghai University of Traditional Chinese Medicine, 358 Datong Rd, Shanghai, 200137 People’s Republic of China; 2 Department of Oncology, Shanghai East Hospital, Tongji University School of Medicine, Shanghai, 200120 China; 3 State Key Laboratory of Oncogenes and Related Genes, Shanghai Cancer Institute, Renji Hospital, Shanghai Jiaotong University School of Medicine, Shanghai, 200032 China; 4 Department of Gastrointestinal Surgery, Shanghai East Hospital, Tongji University School of Medicine, Shanghai, 200120 China

**Keywords:** Circulating tumor cell, Magnetic immunoliposomes, Epithelial cell adhesion molecule, Clinical verifications

## Abstract

Tumor cells with heterogeneity and diversity can express different markers. At present, positive separation of circulating tumor cells (CTC) taking EpCAM as the marker was used in most cases which could be one-sided, while this study successfully prepared four antibody-modified magnetic immunoliposomes (MIL) by using the self-assembled liposome with antibody derivatives. This study aims to explore the separation efficiency and clinical detection feasibility of single or combined use of MIL with multi-tumor markers on different tumors. Captured CTC were stained with CK-FITC, CD45-PE and DAPI, and fluorescence microscope was used for the observation, analysis and calculation. The result indicated that the CTC number positive rate in blood samples of four different magnetic balls on the same patient could be up to 87.5% in 32 patients with 14 different kinds tumors. While the effect of directly mixed separation by four kinds of magnetic balls was not satisfying. It suggested that the MIL of multi-tumor markers could be a powerful tool for CTC separation in application of tumor screening and prognosis.

## Background

Proliferation and metastasis of malignant tumor cells are the key factors resulting in tumor patients' death [1, 2]. At present, the curative effect of tumors is not ideal, mainly due to post-operative recurrence caused by tumor adjacent and distant metastasis [3, 4]. Tumor has been perceived as a systematic disease, and early cancer should be considered as systematic disease even there's no clinical and influential evidence [5]. Considering biological characteristics of tumors, early diagnosis had roused physicians' attention [6, 7]. Physicians and patients had gradually attached great importance to the concept of early screen, which could be helpful for the improvement of curative effect on tumors [8, 9]. It's difficult for traditional tumor diagnosis methods, like imaging monitoring and biopsy which have a certain degree of lagging effect, to achieve early screen [10, 11].

In recent years, circulating tumor cell (CTC) monitoring has become one of the most active fields in cancer research and been applied to the early screen of multiple tumors [8, 12, 13]. CTC examination plays an important role in prognosis prediction, curative effect verification and recurrence monitoring of multiple tumors [14, 15]. So far, CTC examination has been widely applied to multiple malignant tumors [16-18]. For the past decade, researchers from all round the world developed several CTC examination methods and separation techniques, but most of them mainly depend on the surface markers (such as epithelial cell adhesion molecule, EpCAM) of epithelial cells [19, 20]. CTC separation and counting focusing only on positive EpCAM could be one-sided, which could lead to a large amount of tumor cells with other positive markers (such as EGFR positive cells, EMT inverted cells) being ignored, and the sensitivity could be low as well.

Polypeptide magnetic lipid system constructed by lipid materials with similar bilayer structure as the cell membrane could increase the separation efficiency of liver cancer CTC by a wide margin. Based on previous studies [21–23], and focusing on the limitation of the above magnetic immunization positive separation of single EpCAM, this study successfully prepared four antibody-modified magnetic immunoliposomes (MIL), i.e. EpCAM, EGFR, HER-2 and MUC-1, using liposome technique. This study aims to explore the separation efficiency of single use or combined use of MIL with multi-tumor markers on CTC of patients with different tumors so as to find out a more sensitive scheme for the detection of CTC in different tumors.

## Experimental

### Materials

All different tumor cells used in this study were purchased from American Type Culture Collection(ATCC) cell bank. Dulbecco's Modified Eagle Media(DMEM), RPIM-1640 culture solution, fetal bovine serum and trypsin were purchased from Gibco. CD45-PE was purchased from eBioscience; CK-FITC, magnetic grate, dimethyl octadecyl epoxypropyl ammonium chloride(GHDC), Fe_3_O_4_ hydrophobic magnetic nanoparticles (Fe_3_O_4_-HMN) were purchased from Shanghai Shengna Industrial Co., Ltd. DAPI staining fluid was purchased from Beyotime Biotechnology Co., Ltd. EpCAM antibodies were purchased from Shanghai Raygene Biotechnology Co., Ltd. Molecular weight 8000–1400 Da Dialysis bag Purchased from Shanghai Yuanye Biotechnology Co., Ltd. Cholesterol, dichloromethane and other common reagents were purchased from Sinopharm Chemical Reagent Co., Ltd.

### Preparation of antibody derivatives

Take the preparation of Anti-EpCAM antibody derivative as an example. A total of 57.1 μg EpCAM antibody and 100 μg GHDC were dissolved in 3.0 mL phosphate buffered saline (PBS, pH = 7.4), and reacted in the magnetic stirrer at 4 ℃ overnight. The next day, a dialysis bag with a molecular weight of 8000–1400 Da was used for dialysis for 12 h, and the dialysate(ddH_2_O) was changed once every two hours, and it’s freeze-dried after dialysis and antibody derivative EpCAM-GHDC was obtained and weighed. The same method was used to obtain Anti-EGFR-GHDC, Anti-HER-2-GHDC and Anti-MUC-1-GHDC antibodies.

### Preparation of MIL

Weigh 5 mg of DOPC and 5 mg of Cholesterol into two 50 mL three-necked flasks, measure 1.0 mL of Fe_3_O_4_-HMN to ethanol, dissolve in 3.0 mL of CH_2_Cl_2_, and transfer Fe_3_O_4_-HMN/CH_2_Cl_2_ to a three-necked flask. A probe ultrasound equipment was used to conduct emulsification on the round-bottom flask in an ice bath for 6 min. Meanwhile, dissolve 2 mg EpCAM-GHDC in 6 mL ddH_2_O and add to the 3-mouth flasks slowly. After ultrasonic emulsification, a rotary evaporator was used to eliminate the remaining CH_2_Cl_2_. After magnetic separation, the solution was washed for 3 times to obtain magnetic nanoparticles. The same method was used to obtain EGFR, HER-2 and MUC-1 antibody-modified MIL.

### Characteristics and performance of MIL

Polyacrylamide gel electrophoresis (PAGE) was used to detect the antibody content on the surface of magnetic immunoliposomes and confirm the presence of antibodies. Atomic force microscopy (AFM) was used to observe the microstructure of different MIL. PPMS-9 (QUANTUM DESIGN, USA) was used to detect the hysteresis loop of magnetic nanoparticles. An ultraviolet spectrophotometer was used to scan the absorption peak of MIL solution to further confirm the presence of antibodies on the surface of magnetic nanoparticles. The BCA protein quantification method was used to detect the antibody content on the surface of MIL. Zetasizer Nano-ZS 90 (Malvern Instruments Ltd.,UK) was used to detect the diameter and potential of MIL. Nanosight (Malvern Instruments Ltd., UK) was used to verify the diameter of MIL, and diameter change after the combination of MIL and tumor cells was analyzed. A fluorescence microscope (OLYMPUS B × 61, Japan) was used to observe immunofluorescence.

### The experiment on the separation effect of MIL on different tumor cells

Add 30 μL MIL into 7.5 mL PBS solutions containing 100 tumor cells respectively, mix evenly, and conduct magnetic separation for 15 min, discard the supernatant; then add 30 μL of DAPI, 30 μL of CK8, 18, 19-FITC and 10 μL of CD45-PE and mix evenly, stain avoiding light for 15 min; Add to the magnetic separation grate for separation, add 1 mL PBS solution to wash uncombined antibodies, repeat for twice; finally, add 30 μL ddH_2_O and suspend again, when mixed, smear evenly to the center of APES glass slide, observed by fluorescence microscope, take photos, count, and analyze the recovery rate of tumor cells.

### Separation and verification methods of CTC

CTC separation and identification steps of tumor peripheral blood including: Collect 7.5 mL whole blood from tumor patients using anti-coagulation blood collection tube, 1500 rpm centrifuge for 10 min; take pelagic liquid and put it in a centrifuge tube, add isometric PBS buffer(pH = 7.4) and mix uniformly; equally divide into five blood samples: A-E. For blood sample A-D, add 30 μL of four different magnetic nanoparticles respectively, incubate in room temperature for 30 min, blend once for every 5 min; insert EP tube to magnetic separation grate for 15 min of absorption, discard supernatant, and take out the EP tube; conduct magnetic separation wash on the captured CTC for one time using PBS; next, add 30 μL of DAPI, 30 μL of CK19-FITC, 10 μL of CD45-PE, mix uniformly, stain for 15 min avoiding light; after staining, add 1 mL ddH_2_O and conduct magnetic separation for 15 min on the magnetic separation grate, discard supernatant; at last, add 30 μL dd H_2_O in the EP tube for suspending, smear evenly to the center of APES glass slide when mixed, observed by fluorescence microscope, take photos and count. For blood sample E, add 30 μL equal-proportion mixture of four different MIL first, following by the same steps as described above.

### Collection of clinical samples

A total of 32 multi-tumor patients who accepted treatment in our hospital from 2016 to 2017 were collected as subjects, and all the patients were confirmed in clinical diagnosis and pathological examination. The following patients were excluded: those who were allergic to medications; those with other primary tumors; those who were not willing to participate in the experiment; those who didn’t accept radiotherapy and chemotherapy. At the same time, 20 healthy volunteers were enrolled in blood samples. All the subjects signed the informed consent, and this study was approved by the institutional review board of our hospital. Tumor patients and control patients were requested to rest on time the night before blood collection, and 7.5 mL of blood was collected from median cubital vein in the morning of the second day and stored in anti-coagulation blood collection tubes.

### Statistical method

SPSS 19.0 was used to analyze the data. Chi-squared test was used to analyze count variables, statistical significance was set at P < 0.05.

## Results and discussion

### Preparation and characteristics of MIL

**Fig.1:**
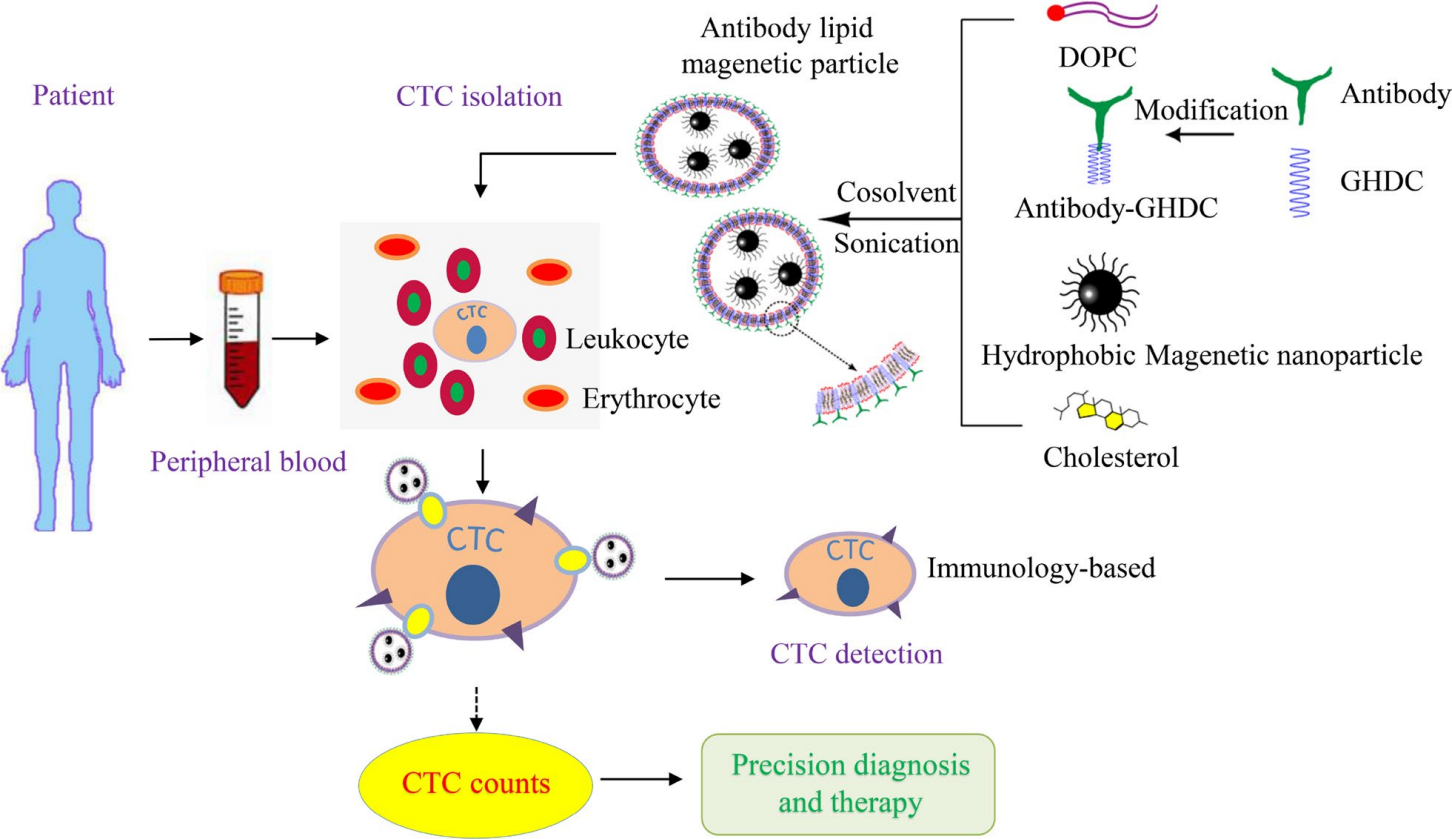
The flow diagram of MIL preparation and CTC detection

MIL preparation and CTC detection flow was shown in Fig. 1. Antibody-GHDC compound derivative was formed by the reaction between antibodies and GHDC, then MIL was made by reversed-phase one-step method combining DOPC, Cholesterol and Fe_3_O_4_-HMN. GHDC modification could increase the antibody content on the surface of immunization nanoparticles and play an emulsification and distribution role in the forming process of magnetic nanoparticles.

**Fig.2:**
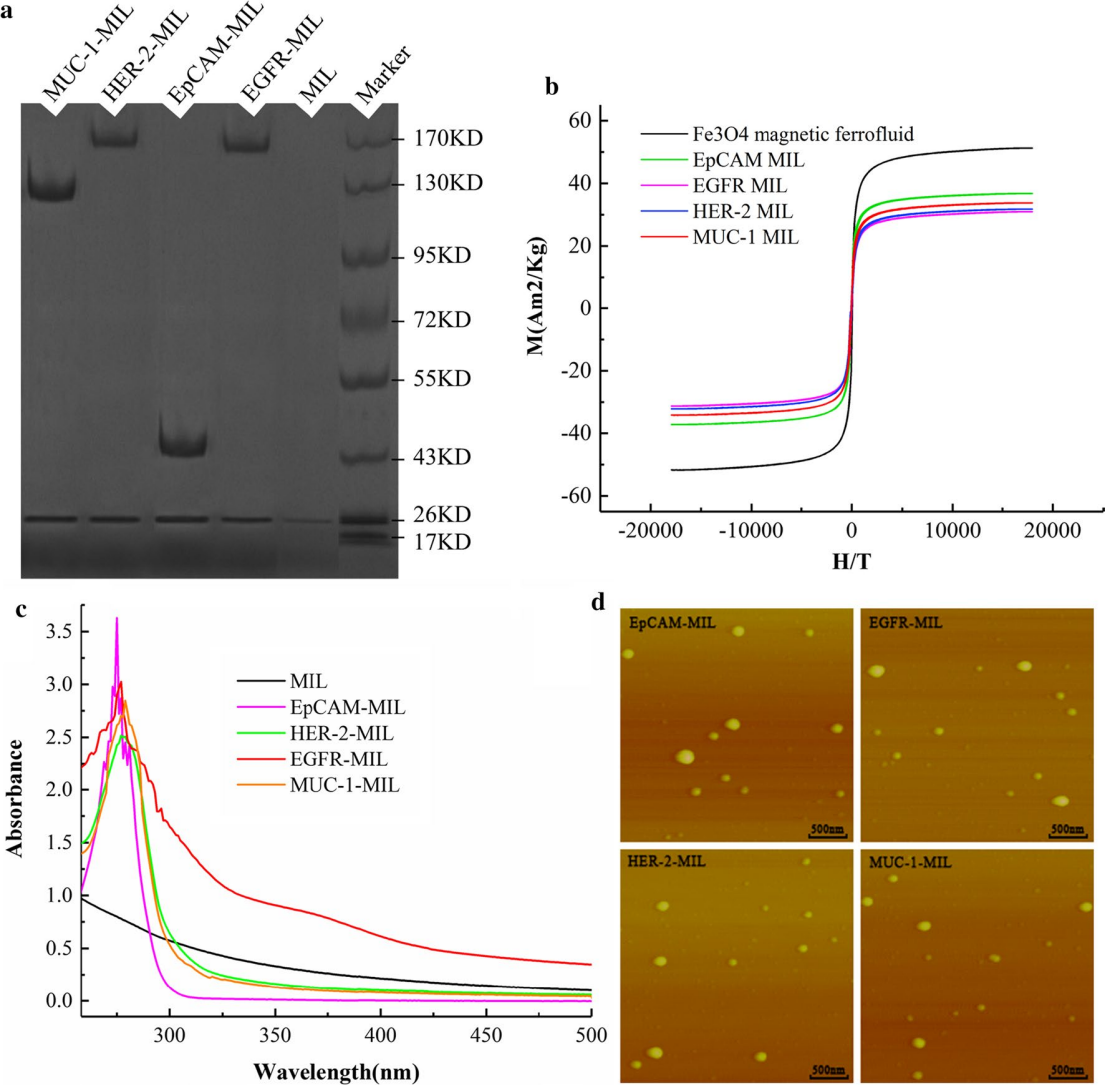
EpCAM-MIL, EGFR-MIL, HER-2-MIL, and MUC-1-MIL Characterization test. **a** Western Blot(WB) results for four magnetic spheres; **b** magnetization curve of four magnetic balls; **c** Ultraviolet test results of four magnetic balls; **d** observation result of four magnetic balls by atomic force microscopy(AFM)

It could be seen from the protein electrophoresis in Fig. 2a that the antibody MIL of different molecular weights has obvious bands, and the MIL without the antibody has no band, which indicates that the antibody and MIL are successfully grafted. Figure 2b demonstrated the magnetic saturation curve of EpCAM, and it's suggested in the experiment result that prepared EpCAM magnetic nanoparticles possessed a high saturation magnetization degree. It could be seen from the figure that magnetic hysteresis was not observed in both Fe3O4-HMN and EpCAM-MIL lines. The hysteresis curves were closed, and residualmagnetic force and coercive force was close to zero within the allowance range of instrument precision, which indicated a superparamagnetic characteristic; the maximum specific saturation magnetization of Fe3O4 magnetofluid was 57.3 emu/g, while the maximum specific saturation magnetization of EpCAM-MIL was 29.9 emu/g, which is about 52% of the pure magnetofluid, the other three kinds of MIL (EGFR, HER-2, MUC-1) had the same magnetic properties as EpCAM-MIL, making MILs can be effectively used for the isolation of tumor cells. The above result suggests that MIL has two obvious advantages comparing with traditional antibody-modified magnetic nanoparticles. On one hand, magnetic nanoparticles coated by lipid can prevent magnetic nanoparticles from oxidation so as to ensure the magnetic separation effect. On the other hand, molecular layer of lipid increases the capacity of antibodies which can increase the recovery rate of CTC. The EpCAM magnetic nanoparticles prepared in this study were highly antibody-modified and possessed a high magnetic intensity, which provides a certain basis for further research on whether it could be applied to screen CTC.

The ultraviolet absorption spectrum of MIL was shown in Fig. 2c. It could be seen from the figure that EpCAM, EGFR, HER-2, MUC-1 magnetic nanoparticles presented obvious ultraviolet absorption peak at 280 nm, magnetic nanoparticles have no peaks. However, as the denaturation of antibodies and influenced by ultraviolet absorption, the absorption peaks of antibody derivatives and immunization nanoparticles at 280 nm were weakened and broadened and shifted a little bit. It’s proved by protein electrophoresis and ultraviolet absorption results that antibodies had been established on the surface of magnetic nanoparticles, and the antibody content on the surface of nanoparticles was 0.1 mg/mg per nanoparticle(BCA quantification method). Observation result of MIL by atomic force microscopy(AFM) was shown in Fig. 2d. As seen in the figure, formed immunization nanoparticles presented an irregular ball structure, with the size of around 200 nm (231 nm), and were not distributed uniformly, and vesicle features of liposome was shown.

### Diameter and other surface characteristics of MIL

**Fig.3:**
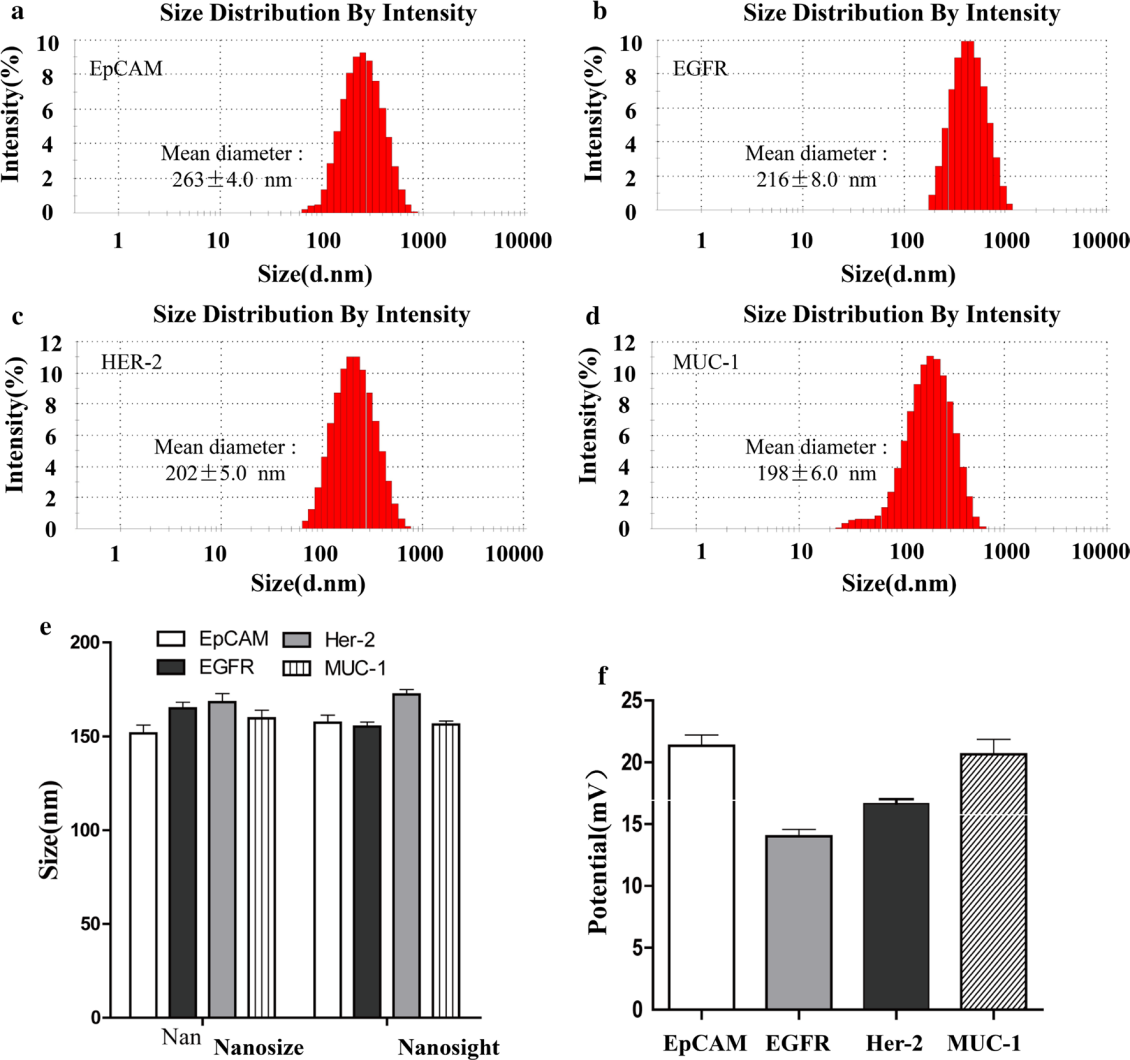
EpCAM, EGFR, HER-2, MUC-1 Particle size and potential test. **a** Particle size distribution of EpCAM nano-lipid magnetic spheres; **b** particle size distribution of EGFR nano-lipid magnetic spheres; **c** particle size distribution of HER-2 nano-lipid magnetic spheres; **d **particle size distribution of MUC-1 nano-lipid magnetic spheres; **e** the size distribution of four kinds of magnetic particles based on nanosize and nanosight; **f** zeta potential of four kinds of magnetic particles

Referring to the preparation method of EpCAM-MIL, three kinds (EGFR, HER-2, MUC-1) of magnetic nanoparticles were prepared. The diameters of magnetic nanoparticles detected by dynamic light scattering method were shown in Fig. 3a–d. There were no differences in the sizes of EpCAM, EGFR, HER-2, and MUC-1 magnetic nanoparticles, which were 263 nm, 216 nm, 202 nm, and 198 nm, respectively. The diameters of magnetic nanoparticles were evenly centered around 250 nm with relatively concentrated distribution interval, presenting consistent specific diameter peaks, which indicated that the preparation technique of lipid magnetic nanoparticles was highly repeatable and stable. The diameters of four different MIL verified by Nanosize and Nanosight were shown in Fig. 3e, which was similar to Fig. 3a–d. And it's suggested by the comparison between the two detection methods in Fig. 3e that the immunization nanoparticles prepared in this study had small diameters and narrow distribution. The result of surface electric potential was shown in Fig. 3f. The surface of four different magnetic nanoparticles presented positive electricity with a quantity of about + 20, and the electricity of four magnetic nanoparticles was not significantly different. The above result indicates that the preparation technique of MIL in this study is highly repeatable, and the diameter of prepared MIL is small with excellent uniformity and centralized distribution peak.

### Comparative analysis on the interaction of MIL and tumor cells

**Fig.4:**
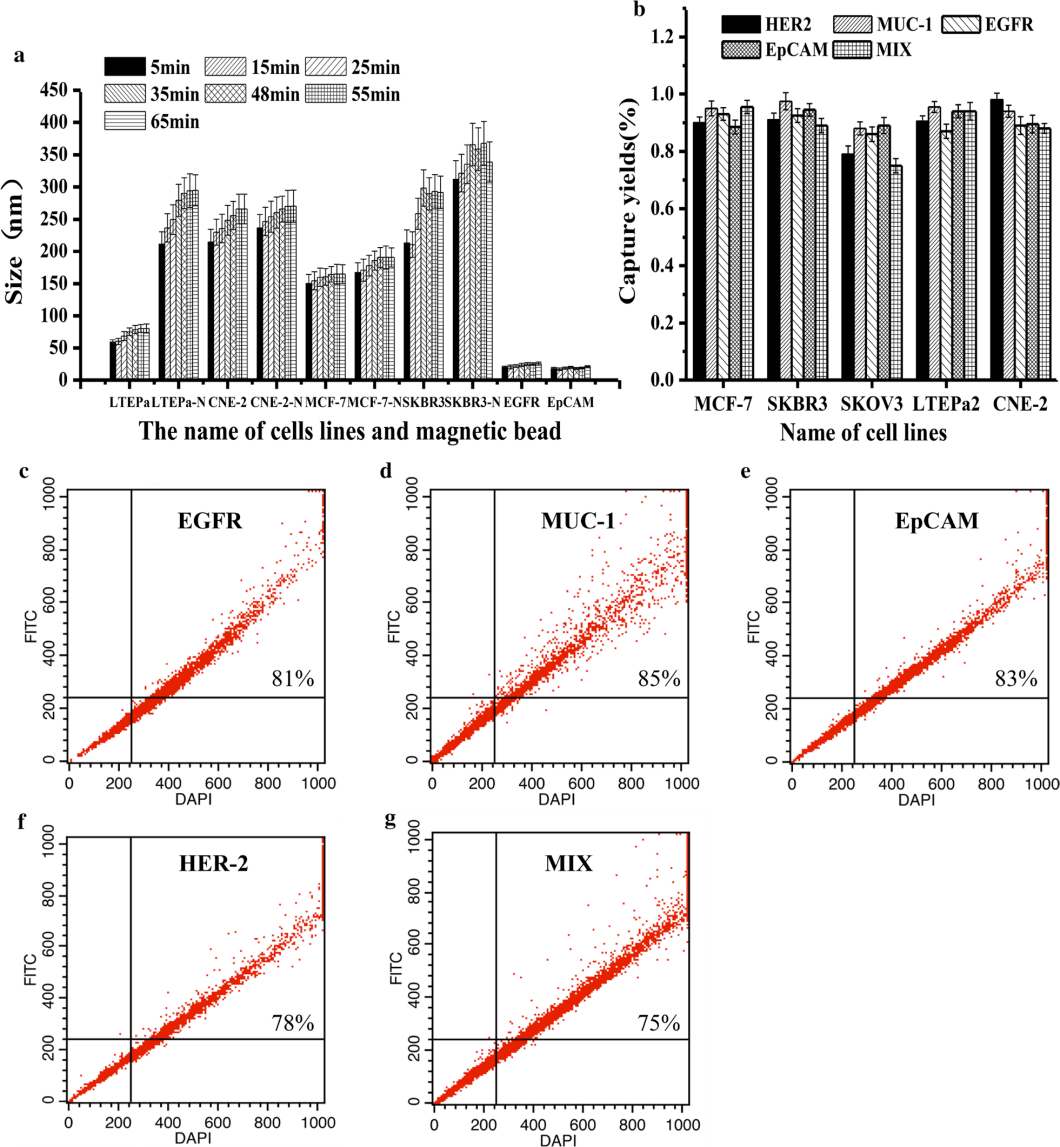
Effect of different lipid magnetic spheres on tumor cells. **a** The particle size of the magnetic particles after binding to the cells; **b **cell capture efficiency of different magnetic spheres; **c** capture efficiency of EGFR magnetic spheres on SKBR3 cells; **d** capture efficiency of MUC-1 magnetic spheres on SKBR3 cells; **e** capture efficiency of EpCAM magnetic spheres on SKBR3 cells; **f** capture efficiency of HER-2 magnetic spheres on SKBR3 cells; **g** capture efficiency of four magnetic spheres on SKBR3 cells

The study on toxicity of MILs to the Growth of Tumor Cells can be see in Additional file 1: Figure S1, four different tumor cell lines were selected, the results show that the MILs constructed in this study have little effect on the proliferation of tumor cells, making the foundation for further study of its function. Nanosight was used to detect the change of diameters of magnetic nanoparticles/cell mixtures in the system after the interaction between immunization magnetic nanoparticles and tumor cells. As shown in Fig. 4a, after the interaction between tumor cells and MIL, the diameters of four different MIL increased significantly, which indicated that prepared immunization magnetic nanoparticles had high affinity to tumor cells. The diameters of magnetic nanoparticles/cell mixtures after 35 min of interaction between magnetic nanoparticles and cells increased lesser, which indicated that as the extend of interaction time, the number of magnetic nanoparticles attached to the surface of cells increased and reached a saturation status at about 35 min. At that moment, increasing the incubation time of magnetic nanoparticles and cells contributed little to the separation efficiency of magnetic nanoparticles to cells. This result was in accordance with the observation result of interaction time of fluorescence-labelled magnetic nanoparticles and cells by confocal microscope. The diameters of magnetic nanoparticles interacting with different tumor cells were significantly different, which suggested that MIL had different capability in the capture of different tumor cells, so the capture efficiency could be varied and cell specific. Laser confocal observation of the interaction of MILs and cell, there is a strong interaction between them, the results are shown in Additional file 1: Figure S2.

### The recovery rate of MIL to different tumor cells

The separation performance of MIL to target cells was analyzed by analyzing the recovery rate of antibody lipid magnetic nanoparticles to 100 counted tumor cells. As shown in Fig. 4b, after mixing with four different magnetic nanoparticles, EpCAM, HER-2, MUC-1 and EGFR could capture four kinds of cells at the same time with a capture rate of up to 80.00% and a maximum capture rate of 97.50%. However, the mixture of four different magnetic nanoparticles didn’t present obviously increased capture rate to cells, which was not statistically different from single kind of magnetic nanoparticles. In conclusion, the antibody lipid magnetic nanoparticles prepared in this study had high affinity to multiple kinds of tumor cells, which can combine tumor cells rapidly and possess a high recovery rate of tumor cells. The separation efficiency of magnetic spheres was also detected by using a flow cytometer (Fig. 4c–g). As shown in Fig. 4c–f, the recovery efficiency of SKBR3 cells by the EpCAM, EGFR, HER-2, and MUC-1 magnetic nanoparticles is 85%, 90%, 86% and 88%, respectively. The combined use of the four magnetic spheres has a capture efficiency of 82% and does not significantly improve (Fig. 4g), this result is consistent with the conclusion of Fig. 4b.

### Morphological observation of circulating tumour cells in clinical blood samples

**Fig.5:**
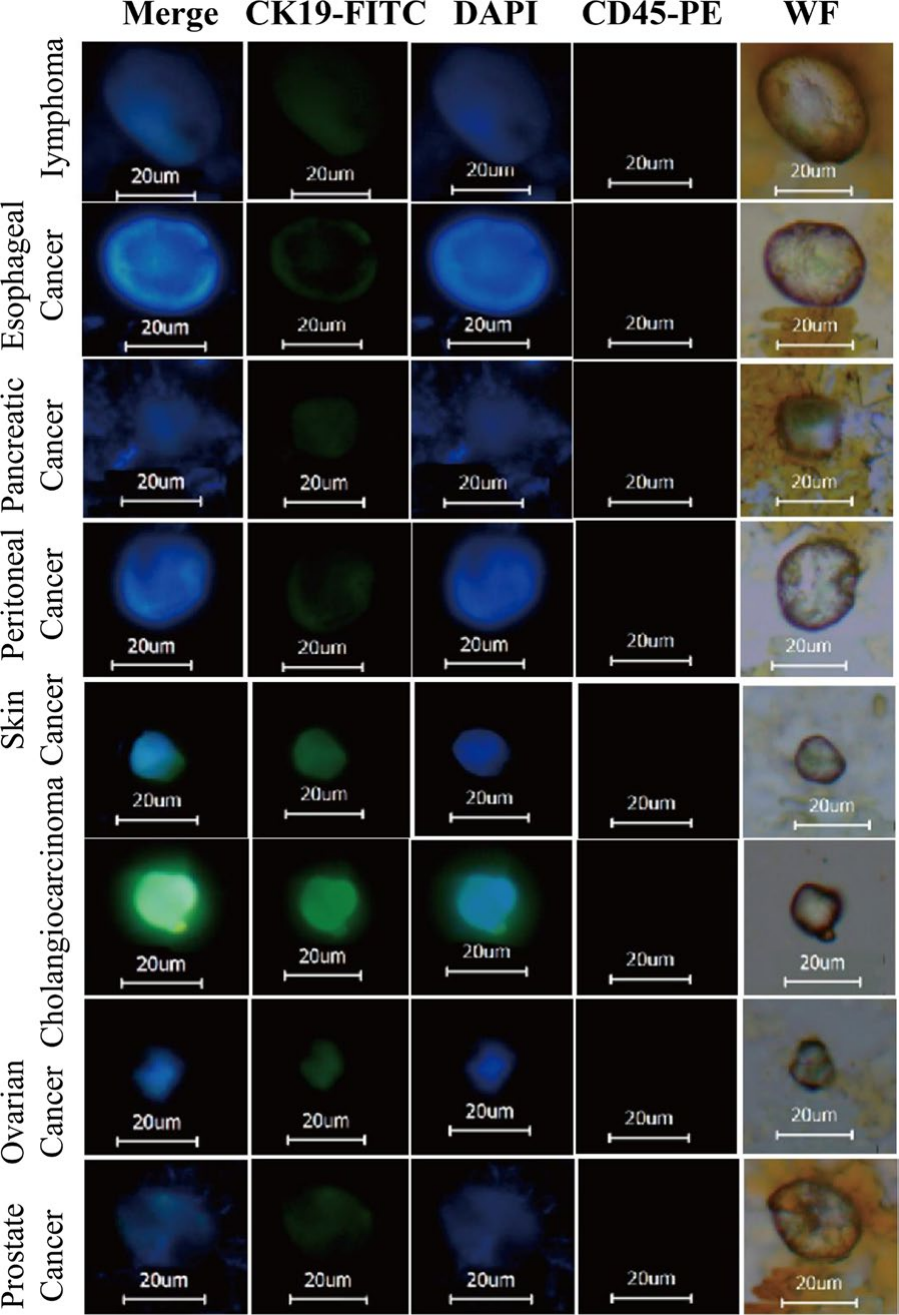
Immunofluorescence observation of CTC in clinical blood samples captured by immunomagnetic particle(Magnification of 400×, white field)

CTC were separated from blood samples of patients with multiple tumors, and were observed by fluorescence microscope after stained by fluorescent antibodies. The imaging result was shown in Fig. 5, cells combined with MIL under white light, with obvious magnetic nanoparticles surrounding the cells. CK8, 18, 19-FITC presented highly positive green fluorescence, DAPI presented highly positive blue fluorescence, CD45 presented negative staining, while cells with a diameter above 8 μm could be determined as CTC. As shown in the figure, the shape of CTC separated from the blood of different tumor patients was similar, presenting as irregular circles or ovals. The green fluorescence of CK8, 18, 19-FITC normally distributed on cell surface. Since the stained CTC were observed in dry state when the cells were collapsed, so the size of blue fluorescence was almost the same as the size of cells.

### Calculation of circulating tumour cells in clinical blood samples

**Fig.6:**
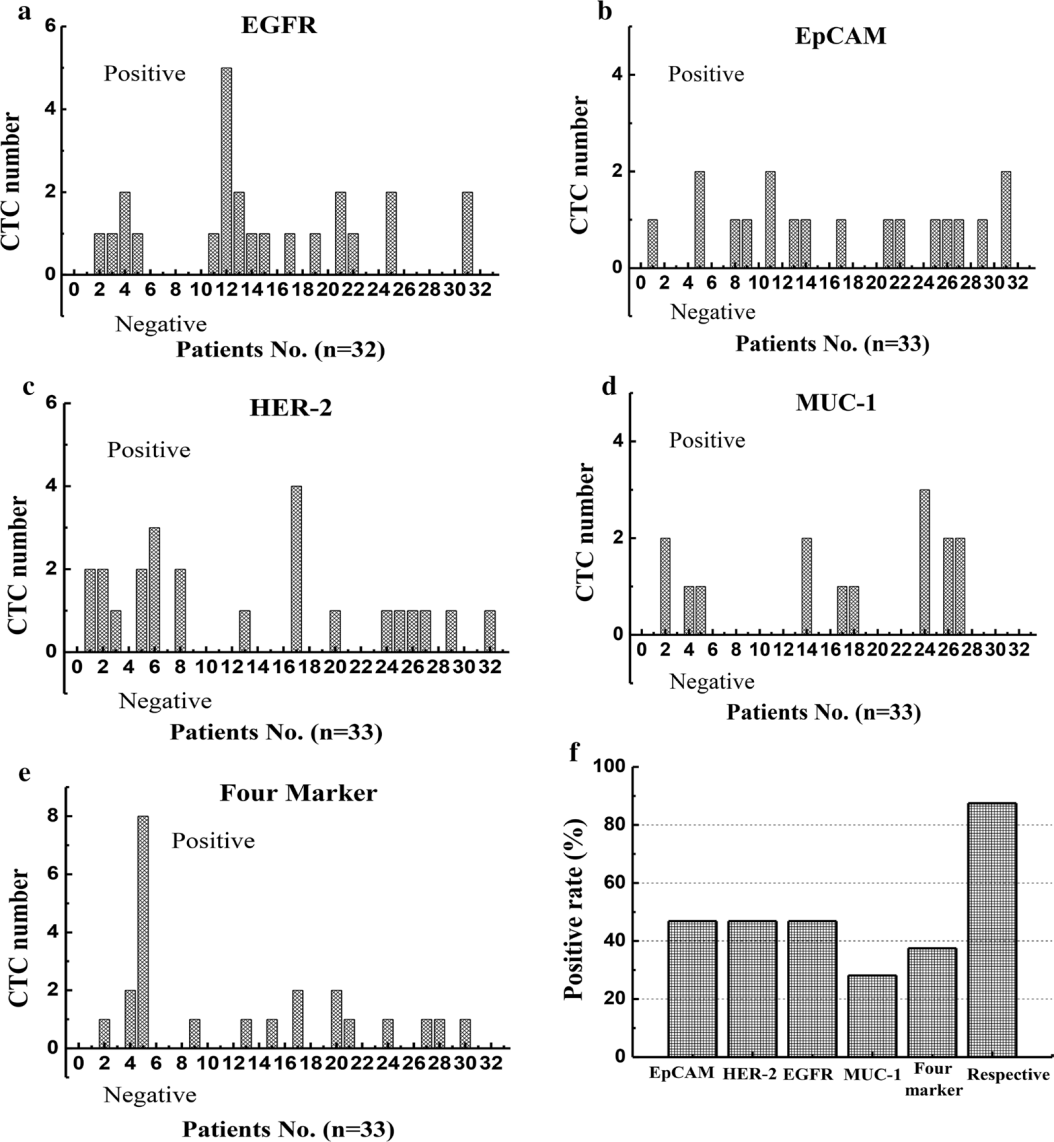
Statistical results of CTC in all patients. **a** EGFR magnetic ball capture CTC statistics; **b** EpCAM magnetic ball capture CTC statistics; **c** HER-2 magnetic ball capture CTC statistics; **d** MUC-1 magnetic ball capture CTC statistics; **e** four magnetic balls capture CTC statistics; **f** positive rate of statistical results

The CTC statistical result of 32 clinical blood samples from tumor patients was shown in Fig. 6a–d respectively indicated the cell number of CTC captured in peripheral blood from 32 patients by four different MIL, and Fig. 6e demonstrated the cell number of CTC captured in peripheral blood from 32 patients by combined use of four different MIL. CTC was not captured in the blood of healthy controls. Fig. 6f demonstrated the ratio of patients with positive CTC result in each experiment plan to the blood samples from 70 patients. EpCAM, EGFR, Her-2 magnetic nanoparticles groups presented high positive rate, above 45%, while the positive rate of the group combining four different magnetic nanoparticles and the positive rate of MUC-1 group was 38% and 30% respectively. However, for single kind of magnetic nanoparticles, the positive rate of CTC separation in peripheral blood of tumor patients could reach 87.5%. The above result suggested that the group with single use of EpCAM, EGFR, Her-2 could generate a better separation effect than the group with combination use of four different magnetic nanoparticles, and the separation effect of MUC-1 group was low. Considering the heterogeneity of tumor cells, in clinical examination, four different magnetic nanoparticles can be applied in the separation and verification of CTC in blood on the same patient respectively.

### Different CTC counts in different tumors and their clinical significance

**Fig.7:**
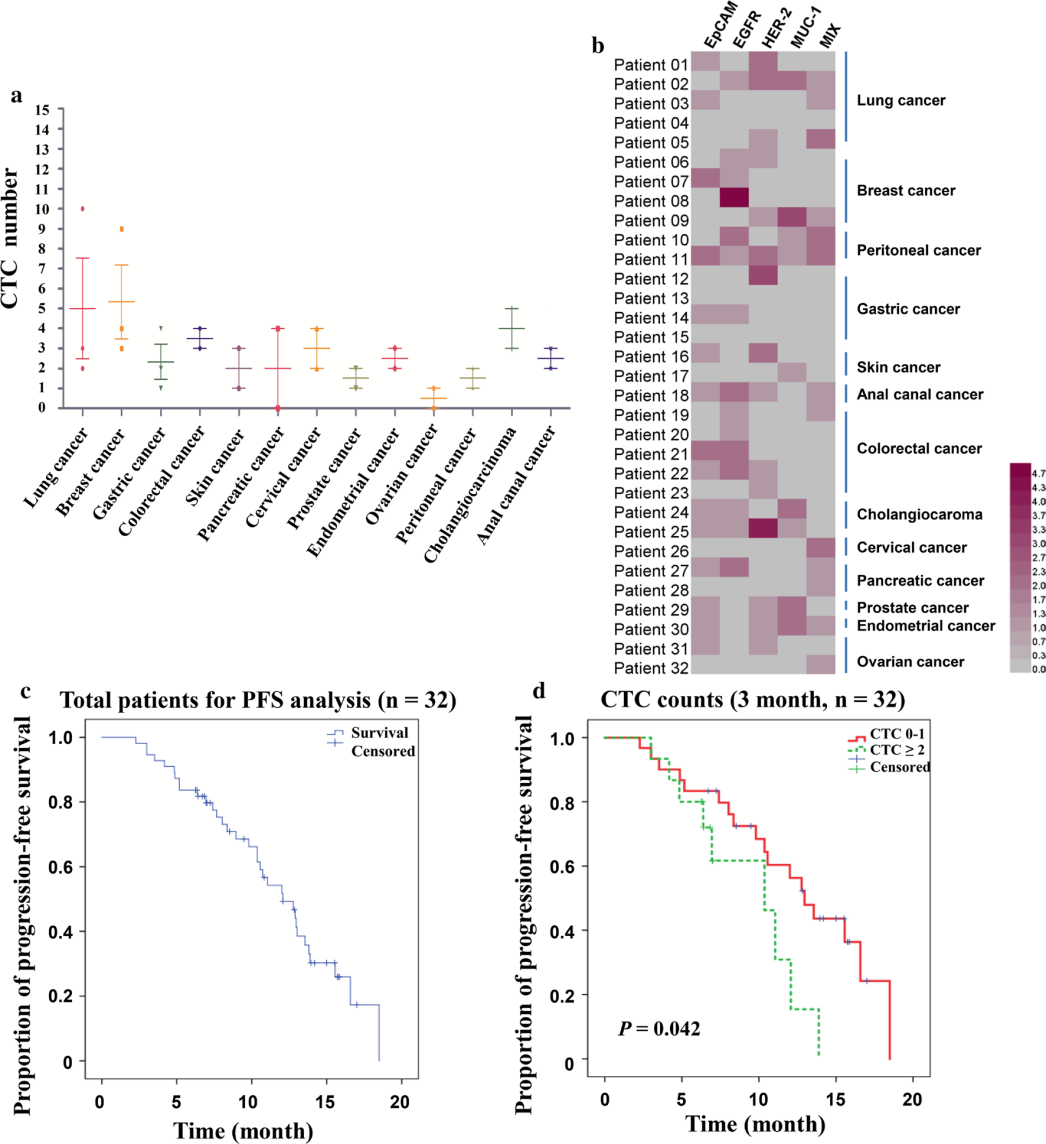
Clinical data of cancer patients. **a **Statistical results of CTC in different tumors; **b **Heatmap display number of CTCs in different tumors; **c **The PFS analysis of total patients; **d **the correlation between the number of CTCs and proportion of progression-free survival

The number of CTCs classified by cancer in 32 cancer patients can see in Fig. 7a. 32 cancer patients are divided into 14 categories according to their cancer types, including lung cancer, breast cancer, stomach cancer, etc. In lung cancer, breast cancer, and liver cancer, the mean number of CTCs showed a high level with 5, 5.5, 6.5, respectively. In other cancers, the number of CTCs showed a relatively low level with mean values below 4. However, CTC can be detected in almost all tumor patients. These results predict that the number of CTCs can be used for cancer diagnosis, but the CTC cutoff value needs to be specifically identified and verified by expanding the sample in different types of cancer patients. The number of CTC captured by four magnetic balls and their mixture in different types of cancer patients is presented in the Fig. 7b, the number of CTCs captured by four magnetic balls and their mixture is different in different tumors. Of course, there are different antigens on the surface of CTCs in different tumors. Choosing the right magnetic ball for different cancer types is necessary to increase the detection rate of CTC in different tumors. The results show that the use of EGFR magnetic beads in breast cancer, colorectal cancer, anal canal cancer and pancreatic cancer has a higher detection rate, while the detection rate of HER-2 magnetic spheres was relatively higher in gastric cancer, all magnetic beads have a high detection rate in peritoneal cancer. The PFS analysis of total patients are shown in Fig. 7c and baseline characteristics of 32 patients was shown in Additional file 1: Table S1. It could be seen from the correlation between the number of CTCs and proportion of progression-free survival in 32 patients in Fig. 7d that a relatively low progression-free survival rate is shown when the number of CTC ≥ 2, which indicated that CTC counts may provide independent and useful prognostic information.

## Discussion

In recent years, more and more studies have shown that circulating tumor cells (CTC) are associated with metastatic recurrence and increased mortality of tumors. The detection, statistics and quantitative studies of CTC have become a research boom. At present, the EpCAM-based CTC detection method is one of the most common CTC detection methods. It is worth noting that many recent studies have shown that CTCs are heterogeneous, including epithelial tumor cells, epithelial–mesenchymal transition (EMT) cells, mixed (epithelial and EMT positive) tumor cells, circulating cancer stem cells (CTSC), and irreversible EMT-positive tumor cells; in addition, the expression of CTC surface protein epithelial cell adhesion molecule (EpCAM) is dynamic. EpCAM-based assays were unable to detect CTC, EpCAM-negative cells, CTSCs, and EMT-positive cells with low EpCAM expression. Therefore, EpCAM-based enrichment in tumor spread may underestimate the importance of CTC, CTSC, and EMT-positive tumor cells, and pure EpCAM may not be a perfect marker for detecting CTC [24, 25].

Adequate evidence suggests that other epithelial CTC markers include epidermal growth factor receptor (EGFR), human epidermal growth factor receptor 2 (HER-2), and mucin 1 (mucin). 1, MUC-1), etc., CTSC surface markers include CD26, CD44, CD133 and CXC chemokine receptor 4 (CXCR4), etc., circulating EMT positive tumor cell surface markers are vimentin, fibronectin, calcium adhesion protein-N and calcium adhesion protein-O [25] and so on. Therefore, future research should be to combine EpCAM antibodies with antibodies to other positive tumor cell surface markers to achieve the best results.

This study prepared four kinds of MIL at the same time, and through the analysis of the preparation and structure properties of MIL, proved that an immunization magnetic lipid nanoparticle system with high recovery rate to target tumor cells could be obtain using direct preparation of antibody liposome by antibody derivatives. By analyzing the number of CTC in blood samples from 32 patients with different tumors collected in each group, and comparing the sensitivity of different CTC separation schemes with different tumor-markers, it’s shown in this study that single use of EpCAM, EGFR, Her-2 and MUC-1 could realize a higher CTC separation positive rate than that of combination use.

## Conclusions

This study provides a feasible plan for high sensitive detection of CTC in tumor patients, It suggested that the MIL of multi-tumor markers could be a powerful tool for CTC separation in application of tumor screening and prognosis, and the improvement of application method could be useful for the precise separation and acute calculation of CTC, which also provides a scientific evidence for early broad screening of tumor patients and for the application of curative monitoring after treatment.

## Supplementary information

**Additional file 1: Figure S1.** Study on Toxicity of MILs to the Growth of Tumor Cells. **Figure S2.** Laser confocal observation of the interaction of MILS and cell. **Table S1.** Baseline characteristics of patients.

## Data Availability

All data generated and analyzed during this study are included in this published article.
