# Supplementary material for: High sensitive detection of circulating tumor cell by multimarker lipid magnetic nanoparticles and clinical verifications
Source: J Nanobiotechnology. 2019 Nov 25;17:116. doi: 10.1186/s12951-019-0548-1 (PMC6876097; doi:10.1186/s12951-019-0548-1)
Supplement: Supplementary file 1 — Additional file 1: Figure S1. Study on Toxicity of MILs to the Growth of Tumor Cells. Figure S2. Laser confocal observation of the interaction of MILS and cell. Table S1. Baseline characteristics of patients. [file 12951_2019_548_MOESM1_ESM.doc]

**High sensitive detection of circulating tumor cell by multimarker lipid magnetic Nanoparticles and Clinical verifications**

**Jingde Chen,^#a^Lin Chen,^#a^ Shibin Du,^b^ Jing Wu,^a^ Ming Quan,^a^ Hua Yin,^c^ Yin Wu,^a^ Xiaofei Liang^*b^ and Hong Jiang^*a^**

*^a^ Department of Oncology, Shanghai East Hospital, Tongji University School of Medicine, Shanghai 200120,China. E-mail: jianghong2046@126.com*

*^b^ State Key Laboratory of Oncogenes and Related Genes, Shanghai Cancer Institute, Renji Hospital, Shanghai Jiaotong University School of Medicine, Shanghai 200032, China. E-mail: xfliang@shsci.org*

*^c^ Department of Gastrointestinal Surgery, Shanghai East Hospital, Tongji University School of Medicine, Shanghai 200120,China.*


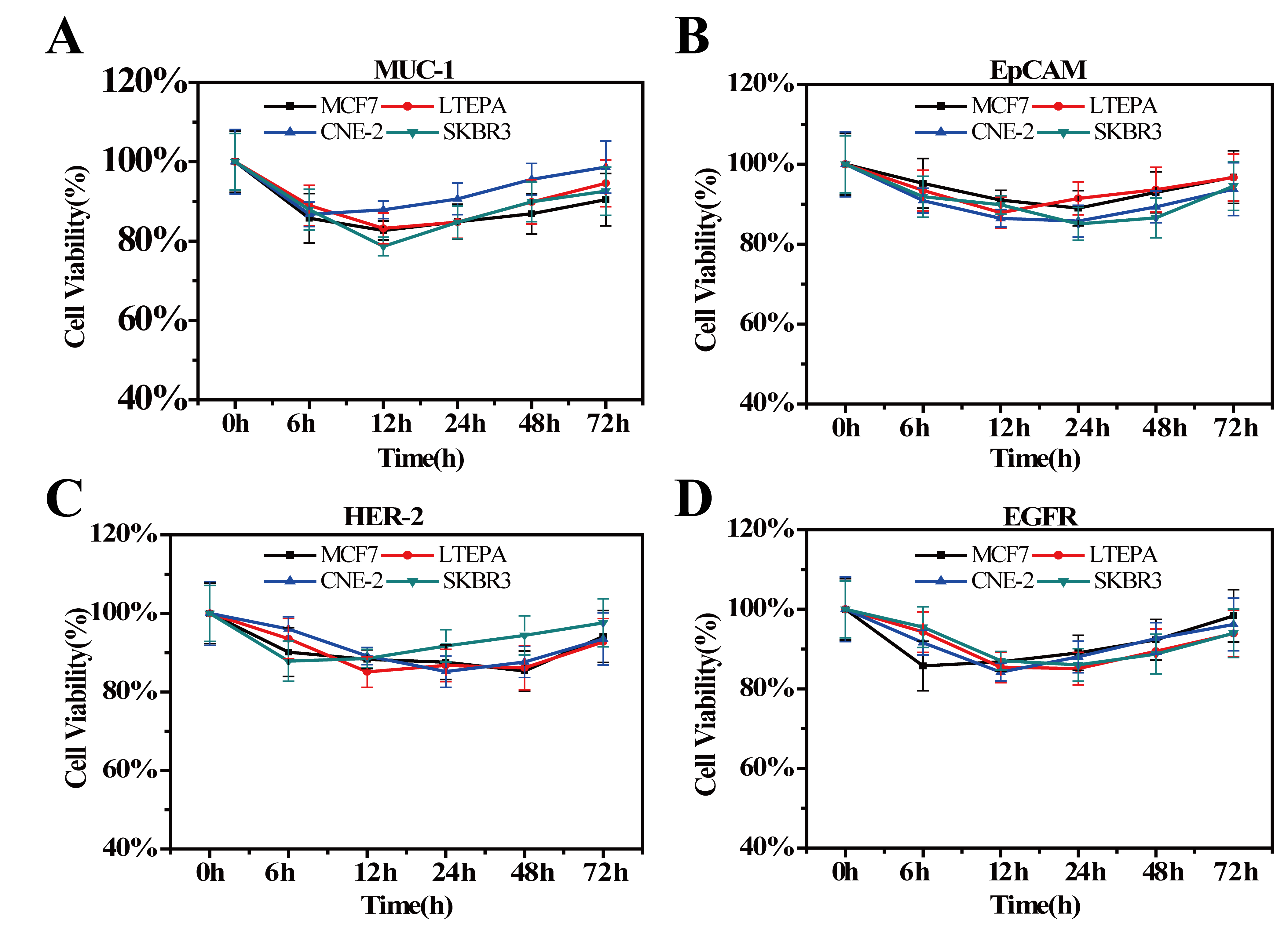


**Figure S1** Study on Toxicity of MILs to the Growth of Tumor Cells


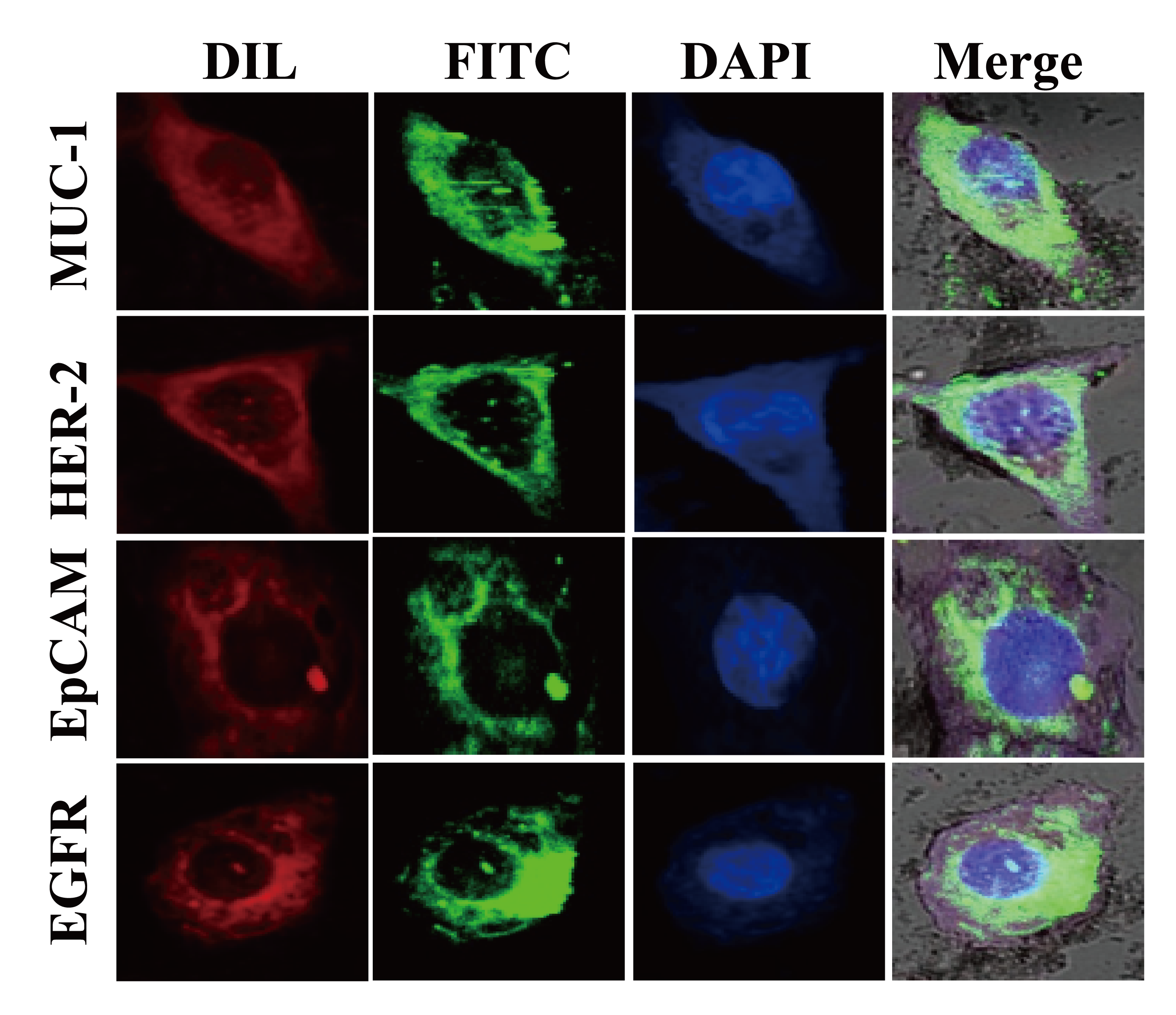


**Figure S2** Laser confocal observation of the interaction of MILS and cell


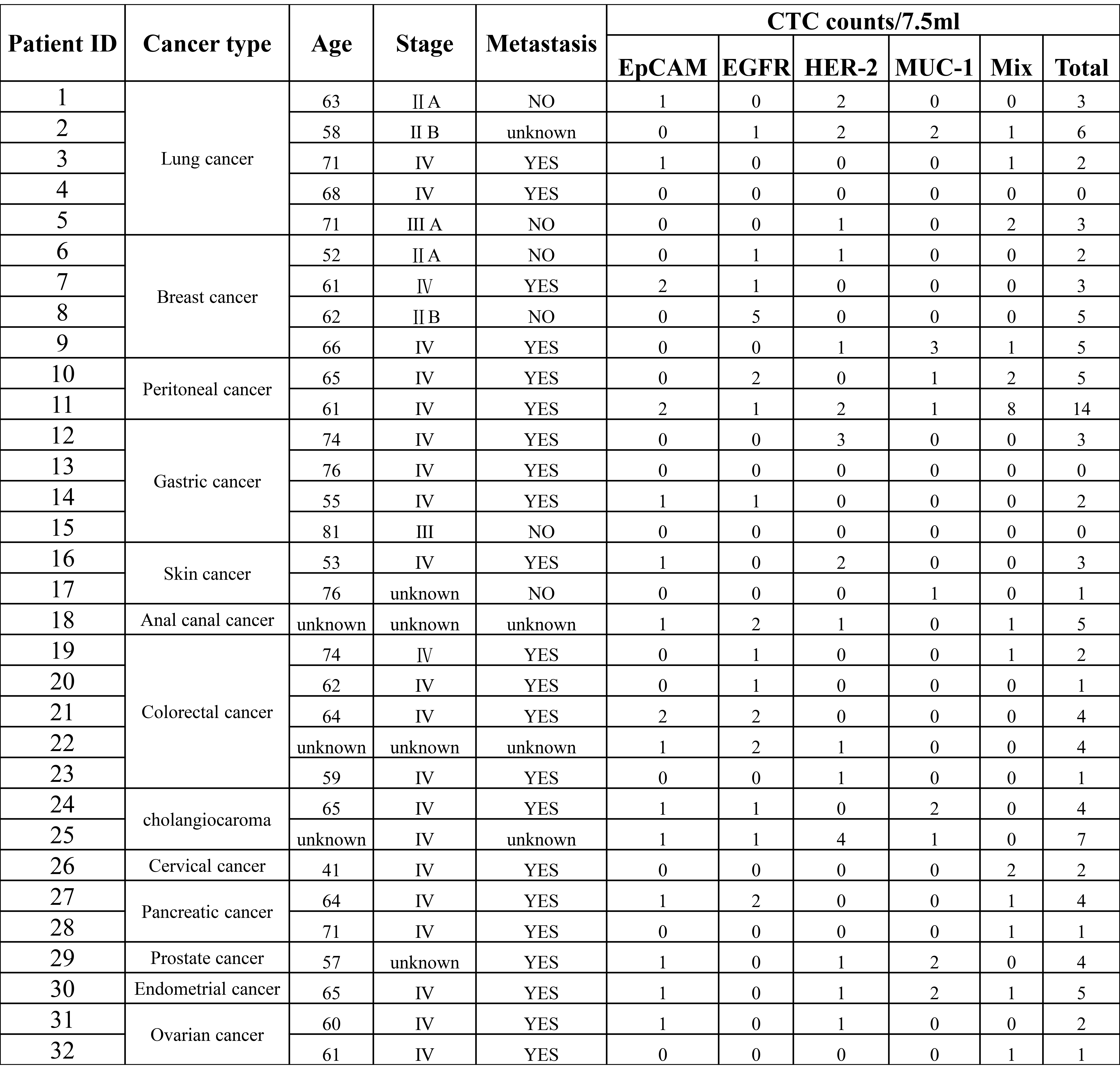


**Table S1** Baseline characteristics of patients
